# Stomach Within a Large Inguinal Hernia

**DOI:** 10.7759/cureus.24783

**Published:** 2022-05-06

**Authors:** Tyler A Grantham, Rajarajeshwari Ramachandran, Swetha Parvataneni, Vinaya Gaduputi

**Affiliations:** 1 Internal Medicine, Staten Island University Hospital, New York, USA; 2 Gastroenterology and Hepatology, The Brooklyn Hospital Center, Brooklyn, USA; 3 Internal Medicine, Geisinger Health System, Lewistown, USA; 4 Gastroenterology, Blanchard Valley Health System, Findlay, USA

**Keywords:** esophagogastroduodenoscopy (egd), gastric ulcers, dilated stomach, inguinal hernia, stomach

## Abstract

We are reporting a case of massively enlarged left inguinal hernia containing the stomach and presenting with coffee ground emesis. Esophagogastroduodenoscopy (EGD) identified a non-ischemic stomach with three small gastric ulcers. The patient opted for non-surgical management.

## Introduction

Inguinal hernias are very common amongst the general population. Men have a lifetime risk of 27%, while women have a 3% risk [1]. Despite how often they present, it is incredibly uncommon to find the stomach within an inguinal hernia. Acquired hernias occur when there is a loss of mechanical integrity of the abdominal wall, allowing abdominal contents to protrude through the defect. Local structures such as omentum, small and large bowel, and genitourinary organs are often displaced into the hernia over time. However, it is very uncommon for a more distant and fixed structure such as the stomach to be included. This paper will highlight a case of a massively dilated stomach found to extend into a chronic inguinal hernia that was evaluated with CT and endoscopy.

## Case presentation

**Figure 1:**
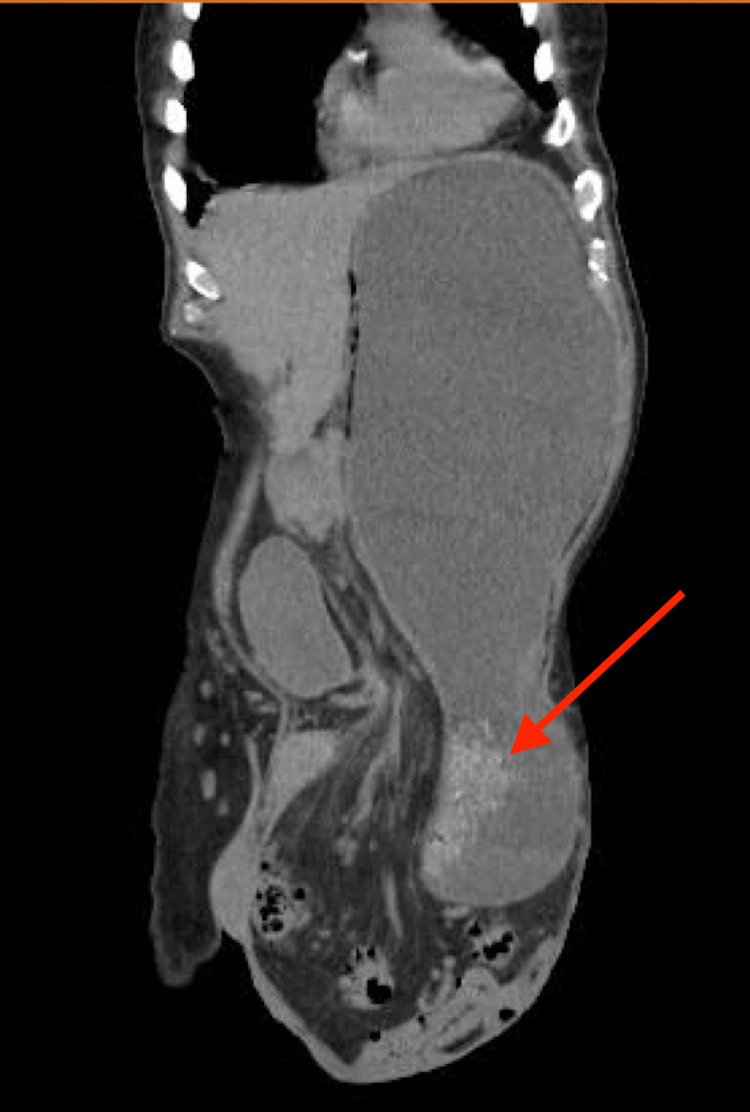
CT Abdomen and Pelvis Demonstrating Stomach in Hernia

An 81-year-old male with alcohol use disorder, presented with few episodes of coffee ground emesis. He did not seek medical attention for more than two decades prior to current presentation. On examination, abdomen was non-tender and with large irreducible, left inguinal hernia with visible peristalsis. Hemoglobin was 13.4 g/dL. Computed tomography (CT) abdomen and pelvis (Figure 1) without contrast showed large left inguinal hernia containing massively dilated stomach filled with fluid, air and retained food, parts of urinary bladder, loops of small and large bowel. No evidence of gastric or intestinal obstruction was seen. Due to the extension of the stomach into the inguinal hernia and the history of coffee ground emesis, patient underwent esophagogastroduodenoscopy (EGD) to exclude ischemia of the herniated stomach, and had friable gastric mucosa, three small gastric ulcers (5 mm each) with stigmata of recent bleeding. The ulcers were treated with epinephrine injection and bipolar cautery. Patient opted for non-surgical management and was discharged home on proton pump inhibitors.

## Discussion

As opposed to bowel, that is more flexible and commonly found in hernias, the stomach is a fixed organ that will typically remain in place. This is because, superiorly, the stomach is secured by the hepatogastric and hepatoduodenal ligaments as well as the gastrophrenic and gastrosplenic ligaments. However, the inferior aspect is secured to the greater omentum by the gastrocolic ligament. It is thought that the force from the omental and bowel weight can slowly pull the stomach downwards by the greater omentum towards the hernia over time [2]. This process will likely only occur when the hernia has been chronically ignored for many years. It is thought that the presence of stomach content in an inguinal hernia is seen less frequently due to the increase in routine repair of hernias over the past several decades [3]. When it does occur, patients often present acutely with signs of gastric outlet obstruction, strangulation, and perforation [4].

Radiological investigations, specifically CT scan, are necessary to visualize stomach within the hernia and evaluate for possible complications [5]. In the setting of a gastric outlet obstruction, they may show gastric distention and possible incarceration. Presence of free air and free fluid in the abdomen are suggestive of perforation. Findings of ischemia from strangulation include wall thickening and abnormal mural attenuation and enhancement [6]. Endoscopy should be performed to visualize a narrowed gastric or duodenal segment and look for the presence of active ulcer. Given the rich collaterals to the stomach, full thickness gastric ischemia is not as common, but is still possible with strangulation. Signs of ischemia on EGD include diffuse or patchy gastric mucosal discoloration, loss of mucosal vascular pattern, diffuse erosions, and/or ulcerations [7].

When the stomach is found within an inguinal hernia, one must evaluate whether surgical intervention is required. Although it can often be managed medically, if there are signs of strangulation or perforation, there should be emergent surgical consultation. Our patient presented with a massively dilated stomach filled with fluid and retained food and was successfully managed without surgical intervention. The patient should receive nothing by mouth and be given bowel rest with fluid resuscitation as appropriate. If the patient has continuous vomiting or substantial abdominal distention, nasogastric decompression and antiemetics are necessary. High dose proton pump inhibitors should be initiated to decrease gastric secretions. In addition, elective surgery should be offered to the patient if possible as they are predisposed to reoccurrence given the weakened abdominal wall.

## Conclusions

Although inguinal hernias are very common, the finding of stomach entrapped within an inguinal hernia remains incredibly rare. Despite the infrequency of the event, it should be considered when a patient presents with a chronically untreated hernia. CT is suggested for diagnosis, and endoscopy can be utilized to guide further management and assess for complication. Conservative management should always be considered, and elective surgery should be offered when appropriate.
